# Erdheim–Chester disease presenting as large vessel vasculitis

**DOI:** 10.1093/rap/rkad074

**Published:** 2023-08-21

**Authors:** Azeem Ahmed, Khin Yein, Shivani Gor, Naim Qamhia, Joya Pawade

**Affiliations:** Rheumatology Department, Great Western Hospital, Swindon, UK; Rheumatology Department, Great Western Hospital, Swindon, UK; Rheumatology Department, Great Western Hospital, Swindon, UK; Department of Pathology, North Bristol NHS Trust, Bristol, UK; Department of Pathology, North Bristol NHS Trust, Bristol, UK

Key message

- Erdheim–Chester disease with vascular involvement can mimic large vessel vasculitis.

Dear Editor, A 62-year-old male presented with critical ischaemia of the lower limbs (Fig. 1A). He had a 4-year history of limb claudication, with a walking distance of 500 metres. Recently, he had described PMR-like symptoms and was noted to have bilateral carotid and subclavian bruits. Large vessel vasculitis (LVV) was diagnosed after CT angiogram demonstrated iliac, subclavian and carotid artery stenosis with aortitis (Fig. 1B). His CRP was 115 mg/l and haemoglobin 71 g/l. His history included diabetes, hypertension, hypercholesterolaemia, pericardial effusion and myocardial infarction. He did not smoke but had multiple risk factors, and claudication symptoms were thought to be related to atherosclerosis.

**Figure 1.**
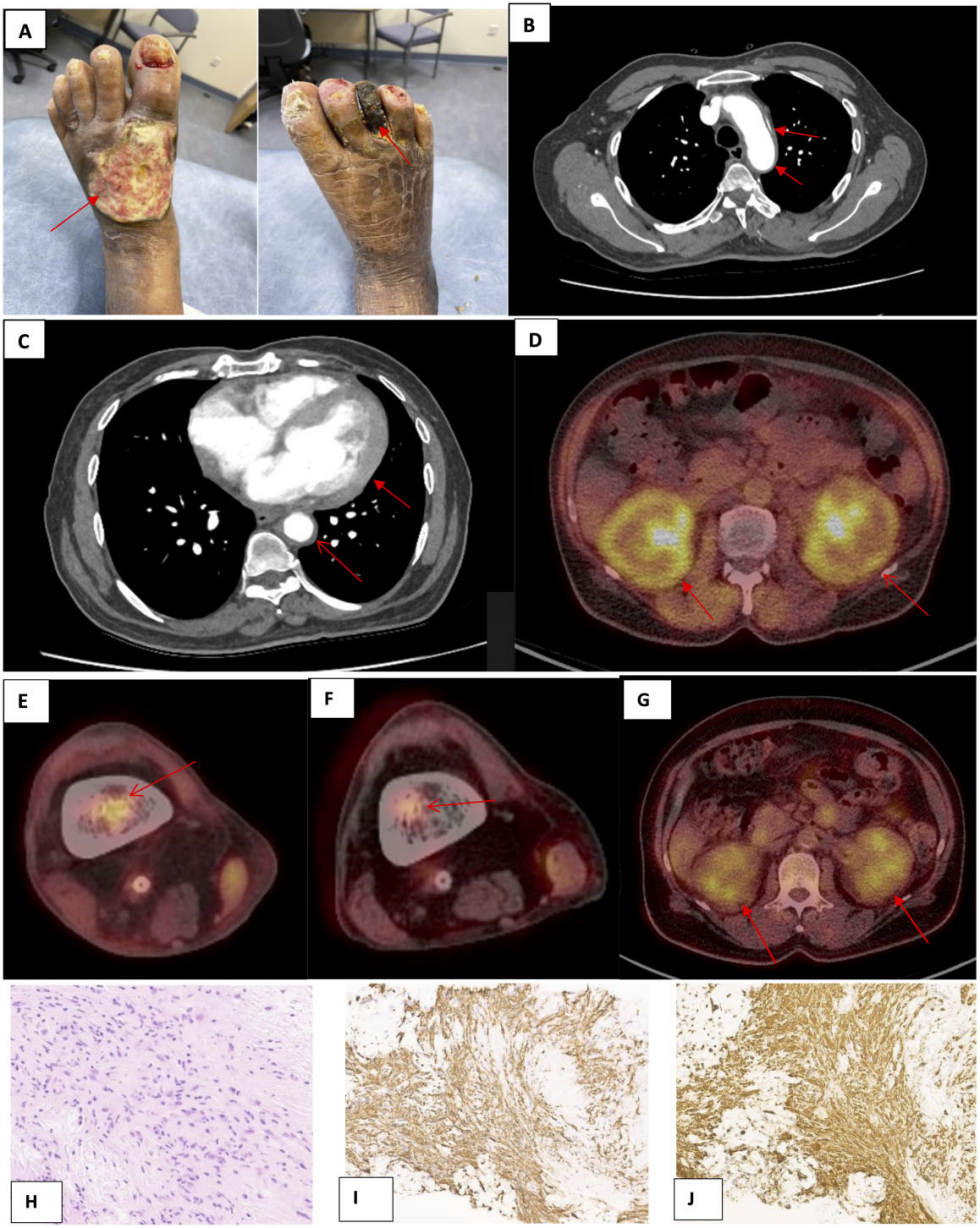
Gangrenous and ulcerated feet with radiological and histological findings. (**A**) Ulcerated and gangrenous feet. (**B**) CT aorta, showing periaortic sheathing (coated aorta). (**C**) Thoracic aorta with pericardial involvement. (**D**) CT-PET, demonstrating hairy kidneys. (**E**) FDG uptake in right distal femur. (**F**, **G**) CT-PET post MEK inhibition, demonstrating reduction in FDG uptake in distal femur and perinephric encasing. (**H**) Core biopsy from a peritoneal lesion, showing proliferation of histiocytic cells, with no significant atypia or mitosis. There is no significant inflammatory cell population within this infiltrate. (**I**, **J**) Histiocytic cells show strong and diffuse expression of CD14 and CD163, respectively. They do not show expression of S100, CD1a or Langerin. Images were taken from scanned slides at ×20 magnification. FDG: Fluorodeoxyglucose; MEK: mitogen-activated protein kinase

With prednisolone 30 mg daily and MTX 15 mg weekly, PMR symptoms improved, and CRP reduced to 32 mg/l. ANA, ANCA, protein electrophoresis, RF and CCP tests were negative.

Repeat CT angiogram found that the thoracic aorta was involved, with infiltration of the pericardium (Fig. 1C). Brightly enhanced kidneys were suggestive of Erdheim–Chester disease (ECD), and increased uptake was confirmed on CT-PET (Fig. 1D). Sclerosis in the distal femora was in keeping with bone involvement (Fig. 1E).

CT-guided biopsy from perinephric tissue demonstrated histiocytosis (Fig. 1H–J). The most common genes tested for in our histiocytic panel included *ARAF*, *BRAF*, *ERBB3*, *HRAS*, *KRAS*, *MAP2K1*, *MAP3K1*, *NRAS*, *PIK3CA* and *PIK3CD*. In our patient, the *MAP2K1* variant was identified, and he was started on an mitogen-activated protein kinase (MEK) inhibitor, trametinib, with good response (Fig. 1F, G).

Erdheim–Chester disease is a rare non-Langerhans histiocytosis with diverse manifestations ranging from indolent localized disease to a life-threatening multisystem disorder. Typical findings include diabetes insipidus, pericarditis, perinephric fibrosis and sclerotic bony lesions [1]. There are ∼1500 known cases in the literature since 1930 [2], with a male predominance affecting 75%, with a mean age of 46–56 years [3, 4].

A series of activating kinase mutations and fusion involving MAPK (RAS-RAF-MEK-ERK) and phosphatidylinositol 3-kinase (P13K-AKT) pathways has been discovered. Melloul *et al.* [5] reported mutations in the MAP-kinase pathway with histiocytosis in 70.4% and *BRAF*^V600E^ mutation accounting for 61.9% of patients. The frequency was ≤80.7% in mixed histiocytosis. Highly sensitive methods, such as digital droplet PCR, can detect *BRAF*^V600E^ mutations in ≤23.6% of patients who were initially negative, emphasizing the need for highly sensitive methods. *MAP2K1* mutations have been noted in 30% and *KRAS*/*NRAS* in ≤27%. The *PI3KCA* mutation activating the AKT-mTOR pathway has been identified in 11%. More recently, activating *CSF1R* mutations were identified.

*BRAF* ^V600E^ mutation in haematopoietic cells result in monocytes circulating in the blood to various organs, where they differentiate into foamy histiocytes. Inflammatory markers are raised in ≤80% of patients, with cytokines including IL-1β, IL-6, IL-8 and CCL-18 [1].

Bone pain occurs in 74–95% of patients, affecting symmetrical long bones and causing osteosclerotic lesions affecting the distal femur and the proximal and distal tibia.

The CNS can be affected in 40–50%, with typical lesions occurring in the cerebellum and brainstem [2]. MRI of the brain can detect retro-orbital inflammation that can mimic IgG4-related disease (IgG4-RD). MRI of the pituitary can detect infiltration that precedes the diagnosis by many years, resulting in diabetes insipidus. Pituitary dysfunction has been observed in ≤91% of patients, with adrenal insufficiency being rare.

Cardiac involvement includes pericardial effusions, ischaemic heart disease and cardiac failure. Thickening of the right atrium creates pseudo-tumours that are best detected by cardiac MRI. Circumferential aortic wall thickening occurs in two-thirds of patients [6].

The lungs are affected in 50% of patients, causing septal thickening, ground-glass appearance or consolidation. Bronchoalveolar lavage reveals macrophages rich in foamy histiocytes [3].

Perinephric infiltration leading to a hairy kidney appearance occurs in 35–65% of patients. This can lead to abdominal pain, with obstructive uropathy leading to renal failure [2].

Xanthelasma-like skin lesions, which are yellow plaques localized to the eyelids, demonstrate multinucleated touton cells on histology [7].

The diagnosis is based on a combination of clinical, radiological, histopathological and molecular tests. CT-PET is preferred; it can detect bony lesions and multi-organ involvement. Dedicated MRI of the brain and heart is required to define the extent of disease. Histology is best obtained from the skin or perinephric tissue because this can be enriched in foamy histiocytes.

Differential diagnosis includes LVV, IgG4-RD and sarcoidosis. IgG4-RD is a fibro-inflammatory disease indistinguishable at the pleura, pericardium, lungs, pituitary, retroperitoneum and large vessels. IgG4-RD lacks long bone involvement, whereas the lacrimal, salivary, tubulointerstitial nephritis and pancreato-cholangitis that occur in IgG4-RD do not occur in ECD. ECD involves the peri-aortic area, with circumferential aortic thickening, and the proximal ureters, whereas IgG4-RD and retroperitoneal fibrosis develop on the anterior-lateral aortic sides and the distal ureters. IgG4-RD differs histologically, with storiform fibrosis and lymphoplasmacytic infiltrates with increased IgG4^+^ plasma cells, but lacks the foamy histiocytes that occur in ECD [8]. LVV lacks other extravascular manifestations of ECD. Symmetrical long bone manifestations are not typically seen in sarcoidosis, but rather focal or multiple bony lesions [1].

Targeted therapy improves mortality by 40% at 3 years [1]. The BRAF inhibitors vemurafenib and dabrafenib have demonstrated efficacy in ≤88% of patients. Treatments targeting MEK with cobimetinib and trametinib are effective in ≤89% of patients.

This case highlights that ECD can mimic LVV. It can be a life-threatening multisystem disorder, with many features overlapping with IgG4 disease, LVV and sarcoidosis. Targeted therapy has vastly improved the prognosis with recognition of the MAPK and PI3KCA pathways.

## Data Availability

No new data were generated in support of this manuscript.
